# Solution Structure and Sugar-Binding Mechanism of Mouse Latrophilin-1 RBL: a 7TM Receptor-Attached Lectin-Like Domain

**DOI:** 10.1016/j.str.2008.02.020

**Published:** 2008-06-11

**Authors:** Ioannis Vakonakis, Tobias Langenhan, Simone Prömel, Andreas Russ, Iain D. Campbell

**Affiliations:** 1 Department of Biochemistry, University of Oxford, South Parks Road, Oxford OX1 3QU, United Kingdom

**Keywords:** PROTEINS, SIGNALING, CELLBIO

## Abstract

Latrophilin-1 (Lat-1), a target receptor for α-Latrotoxin, is a putative G protein-coupled receptor implicated in synaptic function. The extracellular portion of Lat-1 contains a rhamnose binding lectin (RBL)-like domain of unknown structure. RBL domains, first isolated from the eggs of marine species, are also found in the ectodomains of other metazoan transmembrane proteins, including a recently discovered coreceptor of the neuronal axon guidance molecule SLT-1/Slit. Here, we describe a structure of this domain from the mouse Lat-1. RBL adopts a unique α/β fold with long structured loops important for monosaccharide recognition, as shown in the structure of a complex with L-rhamnose. Sequence alignments and mutagenesis show that residues important for carbohydrate binding are often absent in other receptor-attached examples of RBL, including the SLT-1/Slit coreceptor. We postulate that this domain class facilitates direct protein-protein interactions in many transmembrane receptors.

## Introduction

Latrophilins are putative adhesion-class G protein-coupled receptors (GPCR) (Bjarnadottir et al., 2007) widely expressed in the brain (Matsushita et al., 1999). Their physiological ligand or functional roles are unknown, although they have been shown to interact with synaptic scaffolding proteins (Kreienkamp et al., 2002) and are implicated in synaptic neurotransmitter release (Davletov et al., 1998; Willson et al., 2004). Latrophilins are targeted by α-Latrotoxin, the black widow spider venom toxin (Lelianova et al., 1997), and are required for venom toxicity (Mee et al., 2004). The architecture of this receptor is characterized by the presence of a long, extracellular multidomain segment, which includes, in chordates, a rhamnose binding lectin (RBL)-like domain (Ozeki et al., 1991), a hormone binding domain (Perrin et al., 1998), and an Olfactomedin-like domain (Snyder et al., 1991); some of these domains are lost in other phyla, although RBL is always present.

RBL is a relatively rare domain first characterized from sea urchin eggs (SUEL) as a crosslinked homodimer of small, cysteine-rich subunits (Ozeki et al., 1991). RBL proteins show no amino acid sequence similarity to known lectin classes, and do not require the presence of cofactors, such as calcium, for carbohydrate recognition (Ozeki et al., 1991). In all cases characterized thus far (e.g., Hosono et al., 1999; Tateno et al., 1998; Terada et al., 2007), RBL domains cluster through tandem repeats, oligomerization, or both, possibly to increase carbohydrate avidity. The preferred binding monosaccharide of RBL, rhamnose, has no known biosynthetic pathway in animals; hence, it was proposed that egg RBL proteins serve an antimicrobial role, as rhamnose is found in bacterial cell walls (Hosono et al., 1999).

In addition to their presence in the Latrophilin clan, RBL domains exist in extracellular segments of two other metazoan transmembrane proteins, polycystic kidney disease (PKD) 1-like (Li et al., 2003) and a previously uncharacterized single transmembrane protein composed of two extracellular RBL domains, known as C21orf63 in human. A recent *Caenorhabditis elegans* study showed that the latter, termed EVA-1, acts as coreceptor for the SLT-1/Slit neuronal axon guidance cue and influences axon migration functions (Fujisawa et al., 2007). The role of RBL in the ectodomains of these receptors and Latrophilin is not immediately clear, as endogenous rhamnose is rare in animals (Tymiak et al., 1993). One alternative hypothesis is that rhamnose mimics a different endogenous carbohydrate ligand (Hosono et al., 1999), but a noncarbohydrate ligand is another possibility.

Here, we characterize the carbohydrate binding properties of the RBL domain from mouse Latrophilin-1 (Lat-1) GPCR (henceforth simply referred to as RBL), and determine its structure with and without rhamnose. The first structure of this domain class shows that the RBL domain adopts a unique α/β fold with two long structured loops that are important for carbohydrate recognition. Rhamnose is shown to have the highest affinity among various monosaccharides tested, and we were unable to find a higher affinity oligosaccharide ligand in an extensive carbohydrate binding assay. Sequence alignments of transmembrane receptor examples of the RBL domain show that residues important for carbohydrate binding are often substituted, and similar substitutions in mouse Lat-1 RBL abolish monosaccharide binding. We argue that the available evidence suggests that RBL domains recognize noncarbohydrate ligands when in a transmembrane receptor.

## Results

### RBL Characterization

**Figure 1:**
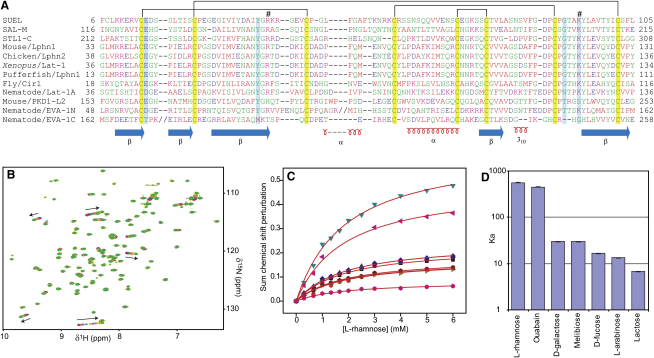
RBL Sequence Alignment and Residue Conservation (A) Amino acid sequences of RBL domains from egg lectins, Latrophilin receptors, and the mouse PKD-1 like 2 and nematode EVA-1 proteins. -N, -M, or -C symbolize N-terminal, middle, or C-terminal RBL domains in tandem repeats. Two nonhomologous sequence insertions in the EVA-1 N-terminal RBL domain (11 residues) and C-terminal domain (seven residues) are omitted and replaced by double slash symbols. Yellow highlight: cysteine residues; cyan: residues involved in carbohydrate binding in mouse RBL; hash symbols: buried charged residues that stabilize the protein loops. Secondary structure elements and the pattern of disulphide formation are shown. (B) Ligand titrations induced chemical shift perturbations in the NMR spectra as indicated by arrows. (C) The extent of these perturbations was plotted against ligand concentration and fitted to extract equilibrium parameters using the equation $\Delta \delta =\Delta \delta_{sat}\left(\left(\left[P\right]+\left[L\right]+K_{d}\right)-\sqrt{{\left(\left[P\right]+\left[L\right]+K_{d}\right)}^{2}-4\left[P\right]\left[L\right]}\right)/2\left[P\right]$, where [P] and [L] are protein and ligand concentrations, respectively, Δδ the shift perturbation measured for each titration point and Δδ_sat_ the perturbation at saturation. Multiple perturbed resonances can be fitted simultaneously to different Δδ_sat_ values for each resonance and a single K_d_ value. Equilibrium parameters (K_a_) measured for different compounds under the same conditions are compared in a logarithmic plot with error bars derived from the fit (D).

The Pfam HMM profile of RBL-type domains includes seven conserved cysteine residues. Genetic constructs of RBL encoding this minimal sequence (mouse Lat-1 residues 47–133) did not, however, produce folded protein, as judged by NMR. An N-terminal extension of approximately 15 amino acids (residues 30–134), including an eighth cysteine, was necessary for folding; additional N-terminal or C-terminal extensions did not appear to contribute to the folded protein core (data not shown). This eight cysteine construct agrees well with previous alignments from tandem repeats of these domains (Hosono et al., 1999; Tateno et al., 1998; Terada et al., 2007; Figure 1A), and with exon boundaries in the gene locus. All cysteine residues were judged to be oxidized based on mass spectrometry. Later structural studies (see below) showed a disulphide formation pattern (Figure 1A) identical to that previously reported for an RBL-type protein from Spanish mackerel eggs (Terada et al., 2007). Analytical ultracentrifugation experiments on RBL (residues 30–134) and larger constructs indicated a monomer in solution (see Figure S1 in the Supplemental Data available with this article online). Inclusion of a ninth cysteine residue through a C-terminal extension did not result in crosslinked homodimers as observed in SUEL (Ozeki et al., 1991), and studies of protein dynamics by NMR showed that RBL remains monomeric, even at high protein concentrations (Figure S2).

Previous studies reported carbohydrate affinity of RBL-type proteins from oligomeric and/or concatemeric variants of this domain (Hosono et al., 1999; Ozeki et al., 1991; Tateno et al., 1998; Terada et al., 2007) with an assay involving carbohydrate inhibition of hemagglutination, which requires multimeric protein forms (Ozeki et al., 1991). Such experiments do not distinguish between direct or apparent affinity effects. We studied the direct interaction of RBL with carbohydrates by monitoring NMR spectral perturbations induced by binding (Figures 1B–1D). In general, RBL binding preferences are similar to those reported for other members of this class (Hosono et al., 1999; Ozeki et al., 1991; Tateno et al., 1998; Terada et al., 2007), with tightest binding for L-rhamnose (K_d_ = 1.8 mM) and significantly less binding affinity for D-galactose, D-fucose, and L-arabinose (Table 1). Other monosaccharide titrations yielded no apparent binding, while titrations with galactose-derived disaccharides showed binding affinities similar to (for melibiose) or less than (lactose) galactose. Physiological saline or 10 mM CaCl_2_ in a tricine buffer did not influence the observed affinities (data not shown). An extended screen performed by the Consortium for Functional Glycomics with 320 mono-, di-, tri-, and tetrasaccharides immobilized on a printed chip array, and fluorescently labeled RBL, did not result in appreciable binding (Figure S3). We were unable to detect the monosaccharide interactions observed in the NMR with the immobilized glycan assay because interactions weaker than 0.1–0.5 mM K_d_ are below the detection limit for this method (David F. Smith, personal communication).

### Description of the RBL Structure

**Figure 2:**
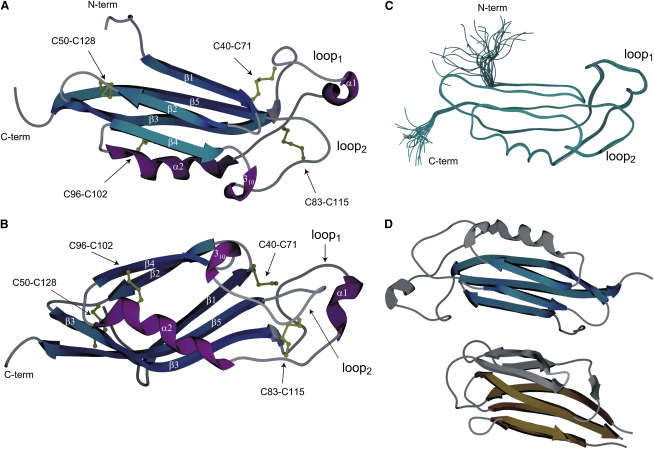
RBL Structure (A and B) Schematic representations of the RBL structure in two perpendicular orientations. The secondary structure elements and loop_1_ and loop_2_ are indicated. Disulphide bridges are shown in gold. (C) The 25 structure ensemble of RBL. (D) Shown in blue and gold are the segments of RBL (top) and nucleoplasmin chaperone core (1K5J, bottom), respectively, that can be aligned with a C^α^ rmsd of 2.6 Å.

The RBL solution structure (Figure 2) was determined from 3476 distance, geometry, and orientation restraints, corresponding to over 35 restraints per ordered residue. A complete analysis of the model characteristics is provided in Table 2. RBL is composed of five β strands (residues 36–41, 45–49, 54–65, 101–105, and 121–130), a single, long α helix (residues 86–96), and two small helical elements (single-turn α-helix residues 75–78 and single-turn 3_10_-helix residues 108–110). The overall fold is that of a β sandwich with two antiparallel sheets (composed of β1, β5, and β3, and β2 and β4, respectively) enclosing the hydrophobic core (Figures 2A and 2B). The two sheets diverge between β4 and β3, and α2 caps the exposed side of the sandwich. Two disulphide bridges stabilize elements of this structural core, connecting the ends of β2 and β5 strands (Cys50–Cys128), and the end of α2 with β4 (Cys96–Cys102).

Unusually for a small protein, RBL includes two long loops (Figures 2A and 2B) connecting β3 and α2 (residues 66–85, loop_1_) and β4 with β5 (residues 106–120, loop_2_). Although these loops do not adopt regular secondary structure, apart from the two single-turn helical elements mentioned, they are well defined in the calculated ensemble of structures (Figure 2C); this agrees with fast-timescale dynamics experiments (Figure S2) that show no significant mobility for these segments. The shorter loop_2_ features a generally conserved proline-rich sequence, PDPCPG, and is in contact with the larger loop_1_. The conformation of these loops is consolidated by two further disulphide bridges connecting the two loops together (Cys83–Cys115) and the end of β1 with loop_1_ (Cys41–Cys71). Importantly, two fully buried charged residues, Arg65 and Lys120, stabilize loop_1_ and loop_2_, respectively, through multiple hydrogen bonds inferred from the structure. Both of these charged residues are highly conserved (Figure 1A and alignment in Terada et al., 2007). Residue-specific substitutions performed at these sites, such as K120A or K120R, yielded proteins exhibiting substantial line broadening and multiple missing resonances in the NMR spectra (data not shown), supporting the idea that these residues have a key structural role.

Structural alignments of known protein models against RBL by Dali (Holm and Sander, 1998) yielded few positive results, none of which included alignments to the two loops. Alignments with other structural tools, such as SSM (Krissinel and Henrick, 2004), did not yield any nonzero probability matches. Removal of the RBL loops from the query model increased the number of aligned proteins in Dali, although the alignment scores remained poor. The five best alignments are summarized in Table S1, and the aligned segments of RBL and the Nucleoplasmin core fragment, the highest scoring protein, are colored in Figure 2D. As indicated, the structure alignment extends only over the β-stranded portion of RBL; none of the aligned proteins features a helix equivalent to α2. Thus, although strictly not a novel fold, the structure of RBL is unique, as evidenced by low structural similarity scores (Z ≤ 3.2). For comparison, structurally well-represented folds of similar size, such as immunoglobulin-type domains, yield similarity scores in excess of 10, and Z scores lower than 2 are not considered statistically significant. Nucleoplasmin core features short conserved loops that assist in the formation of a decameric particle (Dutta et al., 2001). Although these loops are not similar in sequence or structure to the equivalent long RBL loops, it is intriguing to speculate that the RBL loops may assist in protein interactions in the physiological function of this domain.

Marine egg lectins featuring RBL-type domains are primarily found in multimeric forms of tandem repeats, with the exception of SUEL. Based on our RBL structure, there are few linker residues connecting these repeats in known examples—at most, five in CSL3, STL3, and WCL3 lectins (Terada et al., 2007). This would suggest that tandem RBL domains follow closely a beads-on-string model, with limited interactions between domains on the same molecule. Intermolecular interactions are likely present, forming the multimeric species observed; however, our data do not reveal the nature of these interactions, as mouse Latrophilin RBL is monomeric in solution, even under high protein concentrations.

### Carbohydrate Binding to RBL

**Figure 3:**
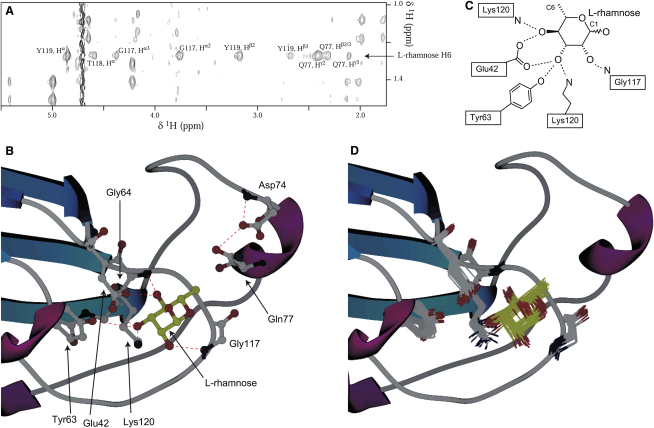
Structure of the RBL/L-Rhamnose Complex (A) Detail from a ^13^C-purged/^13^C-filtered two-dimensional NOESY spectrum acquired on a sample of ^13^C enriched RBL in the presence of excess rhamnose. Residual intraprotein crosspeaks are split to double peaks along the indirect ^1^H dimension due to ^1^*J*_HC_. In contrast, NOE crosspeaks arising from rhamnose appear as single peaks. (B) Detail of the complex structure with rhamnose (gold) and interfacial residues indicated. The hydrogen bonding network formed is denoted by dashed lines. The perturbed Gly64 and Asp74 (through Gln77) residues are also shown. (C) Coordination of rhamnose binding. The C1 and C6 positions are noted for clarity. (D) Interfacial residues and rhamnose in the ensemble of structures.

**Figure 4:**
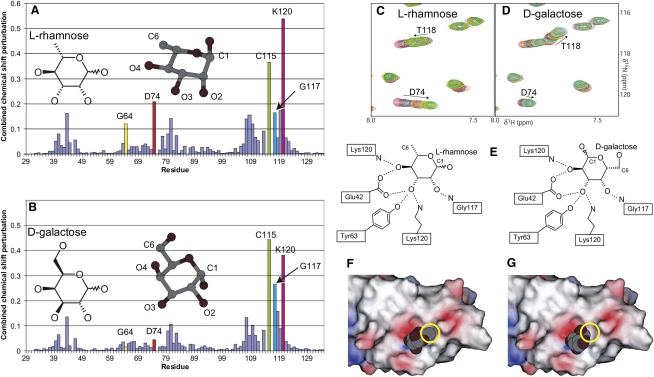
RBL/D-Galactose Binding (A and B) Per residue, combined ^1^H and ^15^N chemical shift perturbations, calculated as $\Delta \delta =\sqrt{{\left(\delta H_{0}-\delta H_{sat}\right)}^{2}+0.04\times {\left(\delta N_{0}-\delta N_{sat}\right)}^{2}}$, derived from RBL titrations with rhamnose or galactose. The structures of the monosaccharides are shown in two- and three-dimensional representations in the α-anomeric form. (C and D) Details from the HSQC spectra overlay of the rhamnose and galactose titrations. (E) Likely binding conformation of galactose compared to rhamnose. (F and G) Electrostatic charge surface representation of RBL in complex with rhamnose or galactose. The rhamnose methyl group and the equivalent position in galactose are indicated in a yellow circle.

Structure determination of RBL in complex with L-rhamnose was based on the average minimized structure of the apoprotein together with intermolecular restraints, as described in the Experimental Procedures. A total of 28 unambiguous and ambiguous distance restraints were used to position the rhamnose residue on RBL. The final 25 model ensemble of structures shows essentially no changes to the RBL domain in the complex (root-mean-square deviation [rmsd] from the apostructure of ∼0.23 Å for the backbone). All intermolecular restraints applied were satisfied within a 0.3 Å limit, yielding a 0.33 Å rmsd for all rhamnose heavy atoms in the ensemble when the RBL backbone was superimposed (Figure 3B). The carbohydrate binding site is located on an exposed pocket primarily formed by loop_2_. In the structure, atoms from two residues of that loop, N and N^ζ^ of Lys120 and N of Gly117, are directly involved in hydrogen bonding interactions (Figures 3C and 3D) with the rhamnose O4, O3, and O2, respectively. The O^ɛ^ of Glu42, from the β1-β2 turn, contribute two hydrogen bonds to rhamnose O3 and O4, and the side chain hydroxyl of Tyr63 a further bond to rhamnose O3. No hydrogen bonds involving the rhamnose O5 or O1 (in either α- or β-anomeric form) could be inferred from the structure; thus, RBL is not expected to be selective for a particular rhamnose anomer. In this configuration, the rhamnose methyl group, H6, occupies a gap between the Gln77 and Tyr119 RBL side chains and the Thr118 backbone atoms (Figure 4F). The side chain orientations of Glu42, Tyr63, and Lys120 do not change substantially in the complex compared to the apostructure. Indeed, in the apostructure, these residues take part in the hydrogen bond network stabilizing loop_2_; rhamnose appears to join this network seamlessly, and complements it by linking the three aforementioned residues to Gly117.

Past studies of RBL domains binding properties suggested that carbohydrate binding depends on the orientation of the O2 and O4 hydroxyls, as these are similar between rhamnose and galactose, while the O3 orientation differs (Tateno et al., 2002). However, our structure of the rhamnose complex indicates that all hydroxyl groups apart from O1 form important hydrogen bonds—no fewer than three in the case of O3. In addition, three-dimensional galactose and rhamnose structures show that the O2 and O4 hydroxyl groups do not occupy the same axial or equatorial positions in both monosaccharides (Figures 4A and 4B). Close inspection of the chemical shift perturbations induced by rhamnose and galactose shows that a number of differences exist. In particular, Gly64 and Asp74 are significantly perturbed in rhamnose, but not in galactose, while the relative perturbations of Cys115, Gly117, and Lys120 are also different (Figures 4A and 4B). Even residues perturbed to a similar extent, such as Thr118, show differences in the manner of perturbation, as seen in Figures 4C and 4D.

We suggest that RBL binds galactose in an inverted configuration around the O5-C3 axis when compared with rhamnose; RBL residues that hydrogen bond to O2 of rhamnose would thus interact with galactose O4, and vice versa (Figure 4E). In this configuration, the inversion-equivalent hydroxyl groups of both monosaccharides occupy the same axial or equatorial positions, rendering rhamnose and galactose identical with respect to the putative hydrogen bonding interactions presented here. Judging from chemical shift perturbation patterns (Figure S4), this type of inverted binding will also be the case for D-fucose and L-arabinose.

This arrangement likely explains the apparent selectivity of RBL domains for rhamnose, as the C6 rhamnose methyl group affords better complementarity with the binding pocket than galactose (Figures 4F and 4G); it also indicates a mechanism for selection of α-linked galactosides. Moieties β linked to galactose would adopt the same equatorial position as rhamnose C6 (Figure 4F); however, due to their large size, this would result in a steric clash with residues on RBL loop_1_. This is consistent with the observed reduction in binding affinity (Table 1) and enhanced chemical shift perturbations in loop_1_ observed in the lactose titration (Figure S4). In contrast, α-linked moieties occupy an axial position and would not interact further with the protein, consistent with the observed identical binding and induced perturbations of melibiose and galactose. Moieties α linked to rhamnose will also occupy an axial position and will not affect rhamnose binding, as seen in ouabain (Tymiak et al., 1993).

### Effects of Residue Substitutions on Carbohydrate Binding

A number of RBL domains, from both transmembrane receptors and free proteins in eggs, feature substitutions of residues involved in carbohydrate binding, especially of residues equivalent to Glu42 and Lys120 in RBL (Figure 1A). Examples of these include the *C. elegans* Lat-1 RBL, the N- and C-terminal RBL domains of EVA-1 (Fujisawa et al., 2007), the RBL of PKD1-like 2 (Li et al., 2003), and individual domains of the SML and STL tandem repeat lectins (Terada et al., 2007). Glu42 in mouse Lat-1 RBL interacts with the carbohydrates by providing two important hydrogen bonds; Lys120 contributes a further hydrogen bond and stabilizes loop_2_. We created substitutions in these two residues of RBL and determined carbohydrate affinity by NMR titrations (Table 1). All substitutions attempted dramatically reduced or completely abolished rhamnose and galactose binding, including conservative mutations, such as E42D, E42Q, and K120R (Figure S5). Thus, we expect that the presence of a glutamate residue at the +1 position from the first cysteine residue is necessary for carbohydrate binding. In the absence of binding, the glutamate-to-aspartate substitution is probably favored over other residues in order to neutralize the buried loop_2_ lysine charge. Similarly, Lys120 cannot be functionally substituted by another positive charge, and is likely optimal for the observed binding.

## Discussion

Carbohydrate recognition domains, or lectins, form a ubiquitous protein class with over 20 lectin families annotated based on amino acid sequence patterns and functional similarities. Lectins have a wide variety of possible roles, including cell adhesion, cell signaling, immune response, host-pathogen interactions, and control of cellular growth (Sharon, 2007). It is generally accepted that many of these roles involve recognition of specific carbohydrate patterns by these domains, typically in the form of oligosaccharides. We have described here what is, to our knowledge, the first structure and monosaccharide recognition mode of RBL, a relatively rare lectin-like domain class. The fold adopted is unique, and carbohydrate recognition involves contacts with the long loop_2_. This is similar to carbohydrate binding by other proteins, such as C-type lectins, that also feature long loops responsible for binding (Zelensky and Gready, 2005). However, in contrast to C-type lectins, the carbohydrate binding loop of RBL is not flexible, and we were unable to find a high-affinity glycan ligand in a solid-state screen, although rhamnose was represented only as a monosaccharide in that assay.

However, rhamnose is a questionable endogenous ligand for RBL domains in extracellular regions of transmembrane proteins. Indeed, there is no evidence that rhamnose binding is the biological function of Latrophilins, rhamnose is found in animals only rarely (Tymiak et al., 1993), and there is no known biosynthetic pathway for it. The direct RBL affinity for rhamnose measured here (K_d_ = 1.8 mM) is weak compared with monosaccharide affinities in other lectin systems (Acharya et al., 1990; Schwarz et al., 1993; Surolia et al., 1996). Simple calculations based on published hemagglutination inhibition data (Hosono et al., 1999) suggest that the K_d_ of rhamnose binding by SUEL is approximately 100–200 μM, 10 times tighter than the affinity displayed by Lat-1 RBL. Therefore, we believe that the single Lat-1 RBL domain would not be sufficient for carbohydrate discrimination, recognition, and attachment. Furthermore, substitutions of residues important for carbohydrate binding, especially Glu42 to aspartate, are common in receptor RBL molecules. This substitution is found in the N-terminal RBL domain of EVA-1, an SLT-1/Slit axon guidance coreceptor (Fujisawa et al., 2007), while the C-terminal EVA-1 RBL domain lacks both the necessary glutamate residue and the conserved loop_2_ lysine residue (Lys120 in RBL) that is involved in carbohydrate binding (Figure 1A). The mouse PKD1-like 2 protein RBL domain also lacks the aforementioned lysine residue; thus, we would not expect it to bind carbohydrates. Sequence alignments of RBL domains from these three transmembrane protein clans in diverse species show that residues important for carbohydrate binding are often absent in one species, but present in closely related organisms. For example, the important glutamate residue is absent in *C. elegans* Lat-1 RBL, but present in *Ostertagia* sp. and *Cooperia* sp. Lat-1. In our opinion, this lack of strict conservation for binding residues in closely related species indicates that carbohydrate binding is not critical for function.

Although the possibility of a yet-unknown binding glycan cannot be discounted, we favor a situation whereby RBL domains in transmembrane proteins, including Latrophilins, recognize noncarbohydrate ligands exclusively or in addition to carbohydrate moieties. These noncarbohydrate ligands could include lipids or, possibly, other proteins through direct interactions. Similar protein-protein interaction functions have previously been demonstrated for C-type lectin domains; for example, in recognition of IgE by the Fc_ɛ_RII receptor (Bettler et al., 1989), Tenascin-R and Fibulin-2 by Lecticans (Aspberg et al., 1997; Olin et al., 2001), and major histocompatibility complex ligands by natural killer cells (Matsumoto et al., 1998). RBL domains may have evolved to accommodate similar interactions with yet-unknown upstream proteins as part of their receptor functions. If correct, our suggestion has implications for possible Latrophilin ligands and the mode of SLT-1/Slit binding by EVA-1.

## Experimental Procedures

### Recombinant Protein Expression and Purification

The RBL domain of mouse Lat-1 (*Mus musculus* Lphn-1) was cloned in a modified pPICZα vector (Invitrogen) for expression in *Pichia pastoris*. Mutant forms of RBL coding for residue substitutions were constructed by a PCR-based method. Approximately 10 μg quantities of these constructs were digested by *SacI* or *PmeI* endonucleases and transformed to *P. pastoris* strain X-33 by electroporation. Successful transformants through genomic integration in the *aox1* locus were selected for initial resistance to Zeocin (Invitrogen), and could be stably propagated in the absence of antibiotic. The proteins of interest were tagged for secretion with the α-factor signal propeptide sequence, and contained a single glycine residue as cloning artifact after posttranslational processing.

Protein expression was performed in minimal media at pH 6.0 with ammonium sulfate and glucose (during biomass growth), or methanol (during induction of protein expression), as sole nitrogen and carbon sources, respectively. Uniform isotopic enrichment was achieved by using ^15^N-enriched ammonium sulfate (Spectra Stable Isotopes), and unenriched or ^13^C-enriched glucose and methanol (Spectra Stable Isotopes) under high-density fermentation conditions. After 5 days of protein expression, cells were discarded and the medium was filtered, followed by 5-fold dilution with H_2_O and adjustment to pH 3.0. The secreted protein was concentrated by retention in a cation exchange column (GE Biosciences) equilibrated in 10 mM sodium citrate buffer (pH 3.0), and eluted with a steep gradient to 2 M NaCl in the same buffer. Protein-containing fractions were pooled, adjusted to pH 6.0, and incubated for 4 hr at room temperature with approximately 1000 U of EndoH endoglycosidase (New England Biolabs). The protein was subsequently dialyzed against a 10 mM sodium phosphate buffer (pH 7.0) and further purified by anion exchange chromatography (GE Biosciences), dialysis against the final NMR buffer, and concentration with Amicon spin columns (Millipore). The final protein concentration was estimated by UV absorbance at 280 nm.

### Protein Characterization

RBL extensively dialyzed against PBS was fluorescently labeled with the AlexaFluor 488 protein labeling kit (Invitrogen). After labeling, free dye was removed by gel filtration chromatography, and the labeling efficiency was estimated as 30%. Approximately 0.1 mg of labeled protein was provided to the Consortium for Functional Glycomics (National Institute of General Medical Sciences/National Institutes of Health) for screening in a printed glycan array chip (version 3.0, 320 glycan targets).

Analytical ultracentrifugation equilibrium experiments were performed at 4°C on 20 μM protein samples in PBS buffer with a Beckman Optima XL-A analytical ultracentrifuge. UV absorbance was monitored at 280 nm. The duration of the run was 48 hr at 25,000 rpm. The data were fit to an ideal monodisperse model with the program Origin (OriginLab).

### NMR Spectroscopy

All experiments were performed at 30°C with home-built spectrometers, with 11.7 T, 14.1 T, 17.6 T, or 22.3 T field strengths. NMR samples consisted of 1–2 mM protein in a 20 mM sodium phosphate buffer (pH 7.0, 2 mM EDTA, 0.1 mM DSS, 0.02% NaN_3_) in 5% or 100% D_2_O, unless otherwise noted. All stages of sequence assignments, protein dynamics, acquisition, and evaluation of structure calculation restraints were performed in a manner analogous to that described previously (Vakonakis et al., 2007). Residual dipolar coupling (RDC) restraints were obtained with a 4% C_12_E_5_ polyethylyne glycol/hexanol alignment medium.

### Structure Calculations

The RBL structure was derived by simulated annealing in torsion angle space starting from an extended conformation, and further refined by a simulated slow-cooling process in Cartesian space with the XPLOR-NIH software package (Schwieters et al., 2003). The rhombicity and anisotropy components necessary for the RDC restraints were determined by grid search with an initial protein structure, and further refined in subsequent calculation iterations. φ and ψ dihedral angle values were predicted with TALOS (Cornilescu et al., 1999) and supplemented, where possible, with values by PREDITOR (Berjanskii et al., 2006). Explicit hydrogen bond restraints were not applied; instead, we used a potential of mean force that conducts a free search for putative hydrogen bonds during the simulation, and optimizes the spatial arrangement of peptidyl backbone units accordingly (Grishaev and Bax, 2004). Only nuclear Overhauser enhancement (NOE), hydrogen bond, dihedral angle, RDC, and ^3^*J*_HNHα_-coupling potential energy terms were used as restraints during simulated annealing. Additional potential energy terms were used during refinement, including a radius of gyration restraint, with a calculated value of 13 Å applied to residues 36–131 (Kuszewski et al., 1999), a conformational database potential term (Kuszewski et al., 1996), and direct refinement against ^13^C^α^ and ^13^C^β^ chemical shifts (Kuszewski et al., 1995). The 25 lowest energy structures (out of 50 calculated) were retained and comprise the final structure ensemble.

Calculation of the rhamnose complex structure was based on the average minimized apo-RBL structure. This was rerefined in the presence of a single sugar residue with the intermolecular NOE distance restraints obtained. Generally, intra-RBL restraints were held invariable, although a small number of RDC and ^3^*J*-derived restraints from RBL residues with substantial chemical shift perturbations were removed. TALOS- (Cornilescu et al., 1999) and PREDITOR (Berjanskii et al., 2006) -derived dihedral angle restraints were recalculated based on ^1^H, ^15^N, and ^13^C chemical shifts upon rhamnose saturation. ^13^C^α/β^ chemical shift-based restraints were also similarly updated. The radius of gyration potential term used during refinement was extended to include the rhamnose residue. Examination of 3D ^15^N-edited NOE spectroscopy (NOESY) spectra acquired in the absence or presence of saturating rhamnose showed a small number of differences in intramolecular RBL crosspeaks. Where applicable, these crosspeaks were reassigned, while repulsive distance restraints were implemented for resonances that disappeared in the complex. Intermolecular distance restraints between rhamnose and RBL were derived from a ^13^C-purged/^13^C-filtered NOESY spectrum (Lee et al., 1994), which selects for NOE crosspeaks between ^12^C-attached and ^13^C-attached protons, acquired in a D_2_O sample of ^13^C-enriched RBL saturated with rhamnose (Figure 3A). Additional intermolecular restraints were obtained by examining the aforementioned 3D ^15^N-edited NOESY spectra of RBL for novel crosspeaks corresponding to rhamnose resonances. Thus, we were able to derive a total of 16 specific intermolecular distance restraints connecting RBL and the H1, H4, H5, and H6 atoms of rhamnose. The RBL ^1^H, ^15^N, and ^13^C chemical shift perturbation data were also used in a manner similar to that utilized in intermolecular docking (Clore and Schwieters, 2003; Dominguez et al., 2003) to give a further 12 ambiguous distance restraints. No explicit intra- or intermolecular hydrogen bonding restraints were used for this calculation, and rhamnose was held at its conformation observed in high-resolution diffraction studies (RCSB codes: 1M7D and 1M7I), which is virtually identical to the idealized models provided by the Hetero-Compound Information Centre. The α-anomeric form of rhamnose was chosen for this calculation because it is three times more populated in solution, although the β form was also considered. A total of 50 structures was generated, and the final ensemble consists of the 25 lowest-energy structures. Galactose modeling to the binding site was based on the conformation found in diffraction studies (RCSB codes: 2NMO and 1OH4).

## Figures and Tables

**Table 1:** Affinity of Carbohydrates to RBL

Compound tested	K_d_ (mM)^a^						
	WT	E42D	E42Q	E42A	E42R	K120R	K120A
L-rhamnose	1.78 ± 0.04	144 ± 3	141 ± 2	NBD	NBD	NBD	NBD
D-galactose	33.6 ± 0.4	—b	—b	NBD	NBD	NBD	NBD
D-fucose	61 ± 1.3	ND	ND	ND	ND	ND	ND
L-arabinose	75 ± 1.3	ND	ND	ND	ND	ND	ND
L-fucose	NBD^c^	ND	ND	ND	ND	ND	ND
D-glucose	NBD	ND	ND	ND	ND	ND	ND
D-mannose	NBD	ND	ND	ND	ND	ND	ND
D-arabinose	NBD	ND	ND	ND	ND	ND	ND
D-glucuronic acid	NBD	ND	ND	ND	ND	ND	ND
N-acetyl galactosamine	NBD	ND	ND	ND	ND	ND	ND
N-acetyl glucosamine	NBD	ND	ND	ND	ND	ND	ND
N-acetyl neuraminic acid	NBD	ND	ND	ND	ND	ND	ND
Heparin	NBD	ND	ND	ND	ND	ND	ND
Ouabain	2.21 ± 0.03	ND	ND	ND	ND	ND	ND
Melibiose	33.3 ± 0.5	ND	ND	ND	ND	ND	ND
Lactose	148 ± 7	ND	ND	ND	ND	ND	ND

NBD = no binding detected; ND = not determined.

a Error reported from curve fitting. Estimates based on multiple independent L-rhamnose titration experiments indicate actual K_d_ errors of approximately ±7%.

b Spectral perturbations detected but were too small to allow K_d_ estimation.

c No spectral perturbations were detected with concentrations of up to 100 mM of compound tested. Based on the average of perturbations observed in other compounds, and the uncertainty in our measurements, we estimate a lower K_d_ boundary in excess of 1 M.

**Table 2:** RBL Structure Statistics and Quality Assessment

		25 Structure Ensemble	Minimized Average Structure	25 Structure Ensemble		Minimized Average Structure	
				Core^b^	Ordered^c^	Core^b^	Ordered^c^
Experimental restraints							
NOE							
Intraresidue (*I − j* = 0)	483						
Sequential (*i − j* = 1)	660						
Short-range (*i − j* < 5)	366						
Long-range (*i − j* ≥ 5)	1212						
Ambiguous	168						
Dihedral angles							
ϕ	79						
ψ	77						
χ_1_	65						
χ_2_	6						
^3^*J*_HNHα_ couplings	88						
^1^*D*_HN_ couplings (RDC)	78						
^13^C^α^, ^13^C^β^ shifts	194						
Total no. of restraints	3476						
Structure Quality							
Rmsds from experimental restraints							
Distance restraints (Å)		0.0159 ± 0.0009	0.0159				
Dihedral angles (°)		0.34 ± 0.04	0.32				
^13^C^α^ chemical shifts (ppm)		1.23 ± 0.02	1.26				
^13^C^β^ chemical shifts (ppm)		1.24 ± 0.02	1.28				
^3^*J*_HNHα_ couplings (Hz)		0.72 ± 0.02	0.69				
^1^*D*_HN_ couplings (Hz)		0.37 ± 0.02	0.38				
^1^*D*_HN_ couplings R factor^a^ (%)		1.2 ± 0.1	1.19				
Distance violations > 0.3 Å		0	0				
Dihedral angle violations >5°		0	0				
Rmsds from idealized geometry							
Bonds (Å)		0.0027 ± 0.0002	0.0028				
Angles (°)		0.610 ± 0.009	0.60				
Impropers (°)		0.54 ± 0.02	0.50				
Ramachandran statistics (%)							
Most favored regions				91.5	77.2	92.5	79.5
Additionally allowed				8.5	21.5	7.5	19.3
Generously allowed				0	0.5	0	0
Disallowed regions				0	0.8	0	1.2
Structure precision^d^							
Backbone atoms (Å)				0.17 ± 0.03	0.19 ± 0.02		
All heavy atoms (Å)				0.64 ± 0.07	0.61 ± 0.05		

a The residual dipolar couplings (RDC) R factor was calculated as suggested by Clore and Garrett (1999).

b Excluding the long loops (residues 65–86 and 106–120) and mobile residues.

c Mobile residues ({^1^H}-^15^N NOE < 0.6) were excluded. Included are residues 36–131.

d Rmsd from the average structure.

## Accession Numbers

The chemical shift assignments of mouse Lat-1 RBL have been deposited in the BioMagResBank under accession number 15553. The structures and structure calculation restraints for the same protein have been deposited in the RCSB Protein Databank under accession numbers 2JX9 and 2JXA for the 25 structure ensembles of RBL and the RBL-rhamnose complex, respectively. The amino acid sequences and numbering schemes used here correspond to the following accession numbers: mouse Lphn1, UniProt Q5U4D5; nematode Lat-1A, Q17505; SUEL, P22031; SAL, Q9PVW8; STL1, Q9IB53; fly Cirl A1Z7G7; mouse PKD1-L2, Q7TN88; nematode EVA-1, Q9XU98; pufferfish Lphn1, Ensembl SINFRUP00000179613; *Xenopus* Lat-1, ENSXETP00000049422; and chicken Lphn2, ENSGALP00000014395.

## Supplemental Data

Document S1. Five Figures, One Table, and Supplemental References
