# Supplementary material for: Solution Structure and Sugar-Binding Mechanism of Mouse Latrophilin-1 RBL: a 7TM Receptor-Attached Lectin-Like Domain
Source: Structure. 2008 Jun 11;16(6):944–53. doi: 10.1016/j.str.2008.02.020 (PMC2430599; doi:10.1016/j.str.2008.02.020)
Supplement: Document S1. Five Figures, One Table, and Supplemental References [file mmc1.pdf]

Structure *16*

Supplemental Data

Solution Structure and Sugar-Binding Mechanism of Mouse Latrophilin-1 RBL: a 7TM Receptor-Attached Lectin-Like Domain

Ioannis Vakonakis, Tobias Langenhan, Simone Prömel, Andreas Russ, and Iain D. Campbell

Table S1. RBL Structural Similarity Search

Protein domain	PDB ID	Backbone RMSD	Aligned residues	Z score
Nucleoplasmin core fragment	1K5J-A	2.6 Å	53	3.2
Complement C1S component fragment	1NZI-A	2.8 Å	54	3.0
Proprotein convertase subtilisin	2PMW-B	3.0 Å	53	2.9
Tetracenomycin polyketide synthesis protein	2GU9-A	2.2 Å	52	2.9
Pullulanase	2FGZ-A	2.9 Å	52	2.9

The five best superpositions of RBL in a structural similarity search by Dali (Holm and Sander, 1998) are summarized here. For each superposition the matching RCSB PDB ID code, backbone RMS deviation, number of superimposed residues and Z score is shown. Z scores lower than 2 are not considered significant.

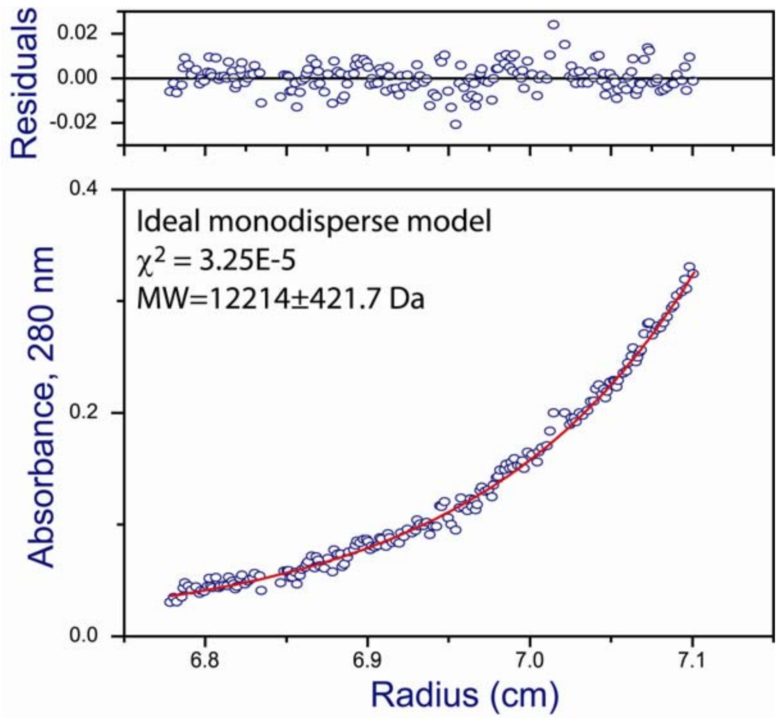

Figure S1. Analytical Ultracentrifugation Equilibrium Absorbance versus Rotor Radius

Data were acquired on ^{15}N-enriched RBL deglycosylated by EndoH, thus retaining an initial N-Acetylglucosamine residue. The 20 μM protein sample in PBS buffer was centrifuged at 25,000 rpm at 4° C for 48 hours prior to data acquisition. Data were fit to an ideal monodisperse model shown as solid line. Residuals of the fit are plotted against rotor radius at the top of the graph. The molecular weight estimated is consistent with a monomer in solution (12339 Da assuming uniform isotopic enrichment and including the N-Acetylglucosamine residue).

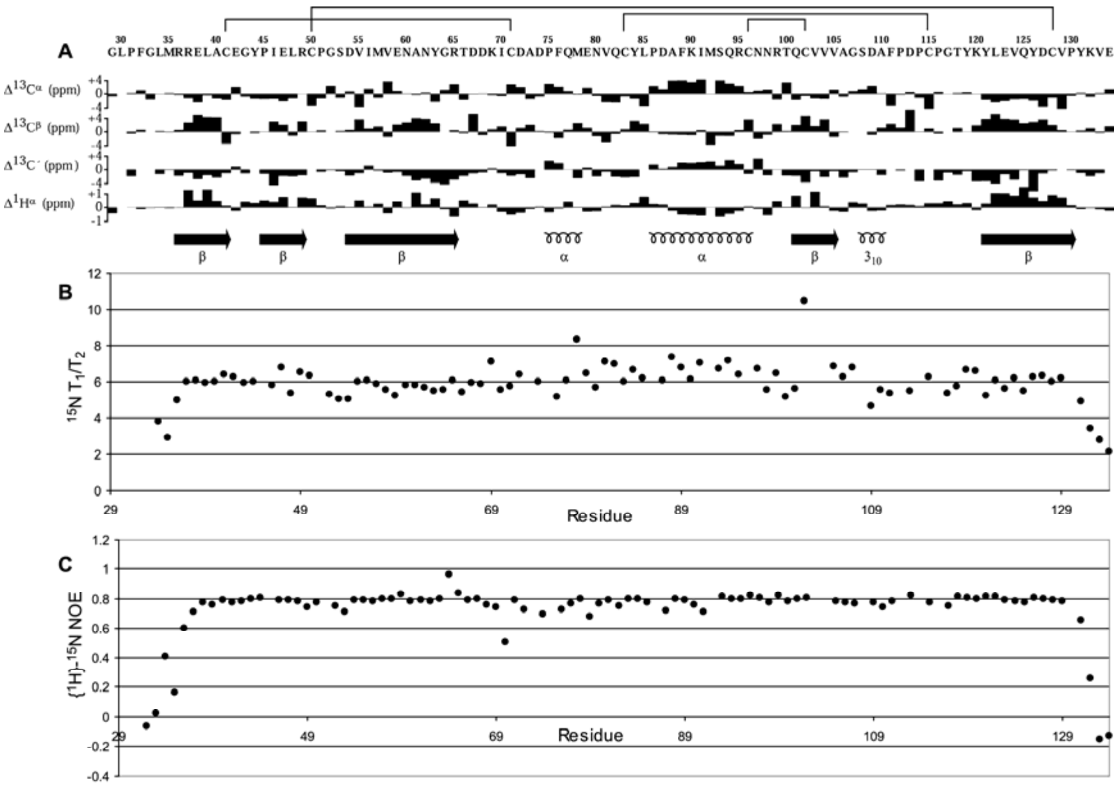

Figure S2. (A) $^{13}C^{\alpha}$, $^{13}C^{\beta}$, $^{13}C'$ and $^{1}H^{\alpha}$ chemical shift differences compared to random coil chemical shifts (Wishart et al., 1995) versus residue number for RBL. Secondary structure elements identified in the final RBL structure are shown, as are the disulphide linkages. (B) ^{15}N T_1/T_2 ratios and (C) heteronuclear $\{^{1}H\}$-^{15}N NOE versus residue number for RBL. Data were collected and analyzed as described previously (Vakonakis et al., 2004) at 30° C and 17.6 T (750 MHz ^{1}H frequency) using a 3 mM ^{15}N enriched RBL sample . The estimated total correlation time (τ_c) under these conditions is ~7 ns which is consistent with a monomeric particle in solution.

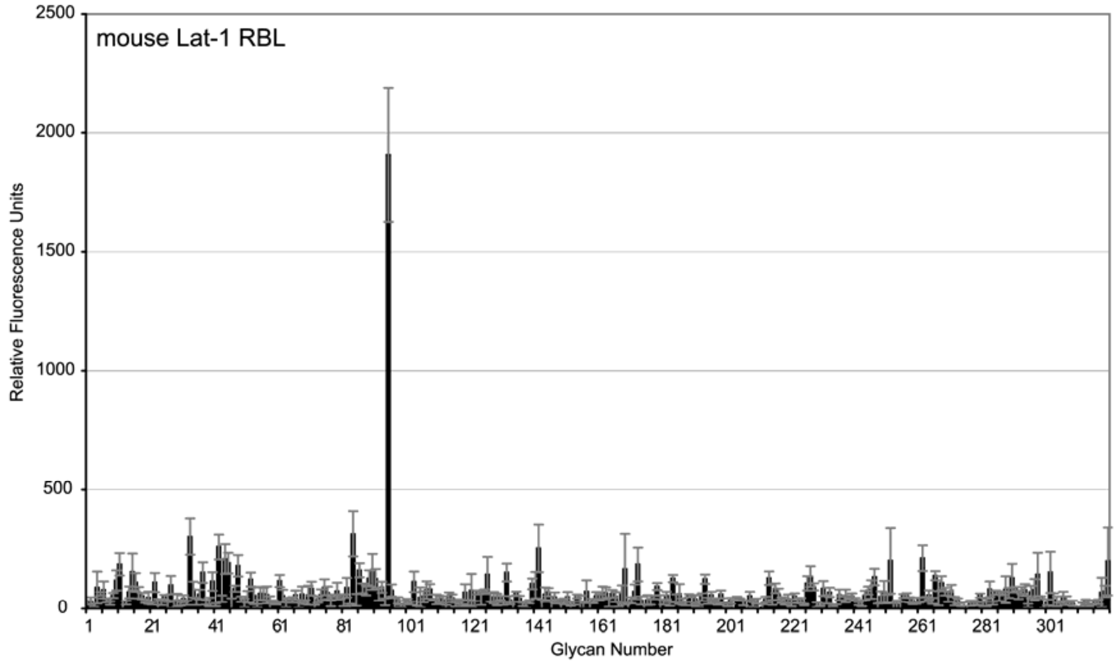

Figure S3. AlexaFluor 488 Labelled RBL Was Tested in Triplicate for Carbohydrate Binding Using an Immobilized Glycan Array Chip (Version 3.0, Consortium for Functional Glycomics, NIGMS/NIH)

Relative fluorescent intensity and estimated errors are plotted against glygan target number. Only one glycan, #95 (Galα1-3(Fucα1-2)Galβ1-3GlcNAcβ-Sp0) showed any interaction for RBL. However, a number of other structurally similar glycans did not yield any binding, and the level of fluorescent signal for glycan #95 is approximately 1/20 of what commonly found in this assay. NMR titrations using glycan #95 as ligand did not show stronger or altered binding compared to galactose. It is thus likely that this binding is the result of a method artefact.

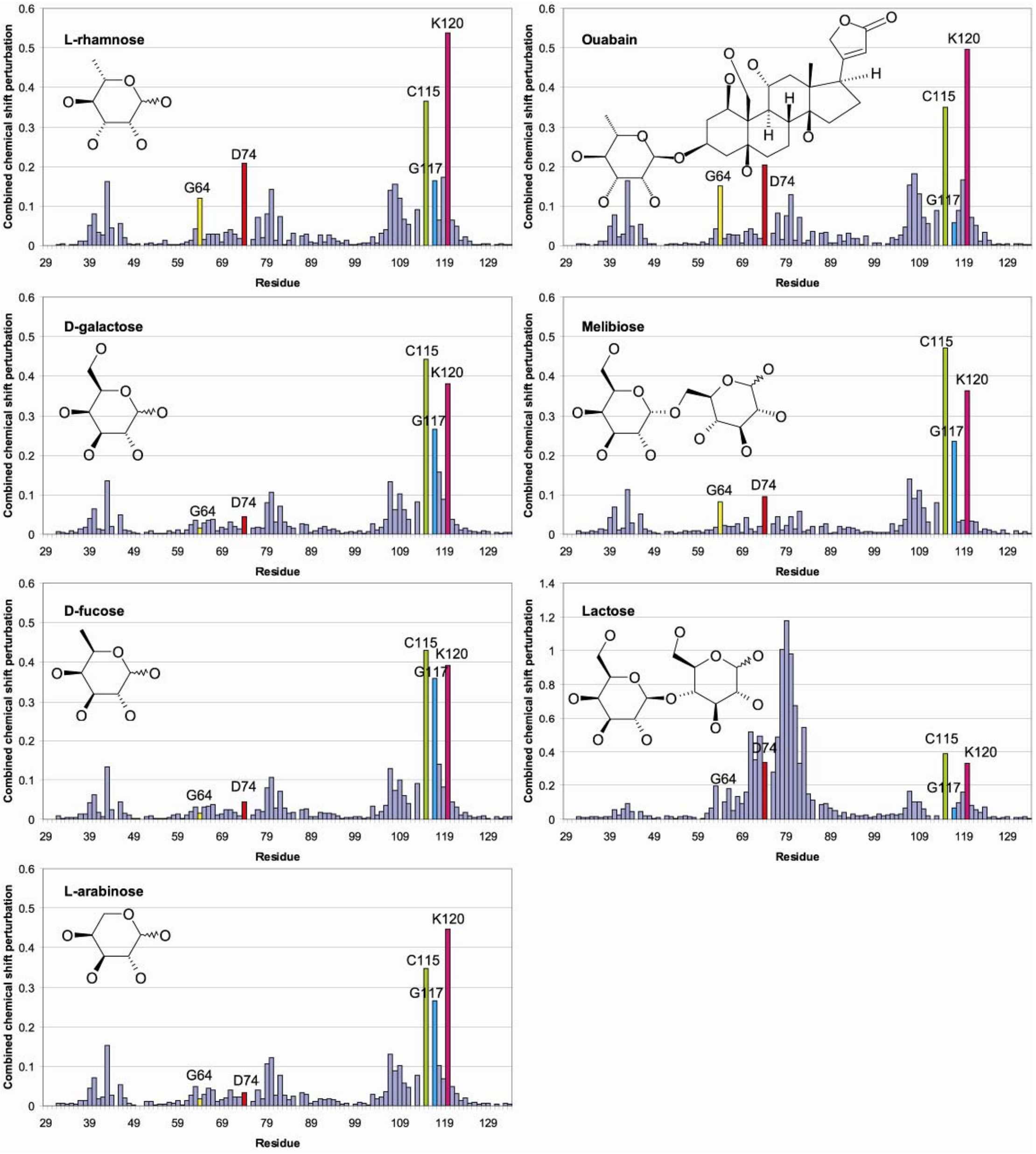

Figure S4. Chemical Shift Perturbations Observed in RBL ^{1}H-^{15}N HSQC Spectra versus Residue Number for Titrations of Monosaccharides or Derivatives

All data were recorded at 30°C using 0.1 mM ^{15}N enriched samples of RBL in a 20 mM sodium phosphate pH 7.0, 2 mM EDTA buffer. ^{1}H and ^{15}N perturbations are reported combined as $\Delta\delta = \sqrt{(\delta H_0 - \delta H_{sat})^2 + 0.04 \times (\delta N_0 - \delta N_{sat})^2}$, where δX_o and δX_{sat} correspond to chemical shifts in the absence or saturating presence of compounds tested, respectively. Chemical shifts at saturation were extrapolated from those achieved in the titrations after data fitting to estimate the percent saturation ratio. In all cases a saturation ratio in excess of 75% was achieved, except for lactose

which was only 50 % saturated. The chemical structures of the compounds tested as shown in the graphs. Note the different relative perturbations of Gly64, Asp74, Cys115, Gly117 and Lys120 between rhamnose and its derivative ouabain (Tymiak et al., 1993), and the remaining compounds. The differences in these chemical shift perturbations are likely due to the presence of the bulky rhamnose methyl group at the Gln77, Thr118 and Tyr119 interface.

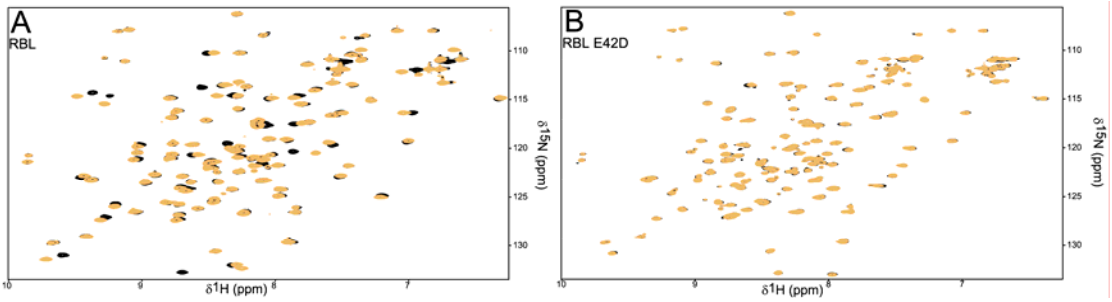

Figure S5. Overlay of (A) Wild-type RBL or (B) E42D RBL ^{1}H-^{15}N HSQC Spectra Acquired in the Absence (Black) or Presence (Gold) of 10 mM L-Rhamnose

Supplemental References

Holm, L., and Sander, C. (1998). Touring protein fold space with Dali/FSSP. Nucleic Acids Res *26*, 316-319.

Tymiak, A.A., Norman, J.A., Bolgar, M., DiDonato, G.C., Lee, H., Parker, W.L., Lo, L.C., Berova, N., Nakanishi, K., Haber, E. et al. (1993). Physicochemical characterization of a ouabain isomer isolated from bovine hypothalamus. Proc Natl Acad Sci U S A *90*, 8189-8193.

Vakonakis, I., Sun, J., Wu, T., Holzenburg, A., Golden, S.S., and LiWang, A.C. (2004). NMR structure of the KaiC-interacting C-terminal domain of KaiA, a circadian clock protein: implications for the KaiA-KaiC interaction. Proc. Natl. Acad. Sci. USA *101*, 1479-1484.

Wishart, D.S., Bigam, C.G., Holm, A., Hodges, R.S., and Sykes, B.D. (1995). 1H, 13C and 15N random coil NMR chemical shifts of the common amino acids. I. Investigations of nearest-neighbor effects. J Biomol NMR *5*, 67-81.
